# Response Surface Methodology to Optimize the Isolation of Dominant Volatile Compounds from Monofloral Greek Thyme Honey Using SPME-GC-MS

**DOI:** 10.3390/molecules26123612

**Published:** 2021-06-12

**Authors:** Marinos Xagoraris, Alexandra Skouria, Panagiota-Kyriaki Revelou, Eleftherios Alissandrakis, Petros A. Tarantilis, Christos S. Pappas

**Affiliations:** 1 Laboratory of Chemistry, Department of Food Science and Human Nutrition, Agricultural University of Athens, 75 Iera Odos, 11855 Athens, Greece; mxagor@aua.gr (M.X.); stud15080@aua.gr (A.S.); p.revelou@aua.gr (P.-K.R.); ptara@aua.gr (P.A.T.); 2 Laboratory of Quality and Safety of Agricultural Products, Landscape and Environment, Department of Agriculture, Hellenic Mediterranean University, Stavromenos, PC 71410 Heraklion, Crete, Greece; ealiss@hmu.gr

**Keywords:** thyme honey, response surface methodology, optimization, volatiles, solid-phase microextraction

## Abstract

This study aimed at an experimental design of response surface methodology (RSM) in the optimization of the dominant volatile fraction of Greek thyme honey using solid-phase microextraction (SPME) and analyzed by gas chromatography-mass spectrometry (GC-MS). For this purpose, a multiple response optimization was employed using desirability functions, which demand a search for optimal conditions for a set of responses simultaneously. A test set of eighty thyme honey samples were analyzed under the optimum conditions for validation of the proposed model. The optimized combination of isolation conditions was the temperature (60 °C), equilibration time (15 min), extraction time (30 min), magnetic stirrer speed (700 rpm), sample volume (6 mL), water: honey ratio (1:3 *v*/*w*) with total desirability over 0.50. It was found that the magnetic stirrer speed, which has not been evaluated before, had a positive effect, especially in combination with other factors. The above-developed methodology proved to be effective in the optimization of isolation of specific volatile compounds from a difficult matrix, like honey. This study could be a good basis for the development of novel RSM for other monofloral honey samples.

## 1. Introduction

Since the Covid-19 pandemic hit global reality, an increased global demand for nutraceutical foods that are used to shield the body against viruses is observed. In the same period, according to the Hellenic Statistical Authority (ELSTAT) and Pan-Hellenic Center for Export Research and Studies (C.E.R.S.), food international exports are high mainly in fruits, vegetables, and, secondarily, honey [1,2]. Honey is a naturally sweet, functional food with various nutritional and protective phytochemical compounds [3,4]. Greek thyme honey is derived from *Thymbra capitata* L. (syn. *Coridothymus capitatus* L., *Thymus capitatus* L.) of the Lamiaceae family. It is probably the most popular, delicious, and high quality Greek honey variety, with extraordinary organoleptic characteristics, especially due to its rich aroma profile. Also, it is recognized for its commercial value in international markets and is greatly appreciated by consumers.

Honey volatile compounds may differ depending on the plant species from which nectar or honeydew secretions are collected but also from handling of the bees or the beekeeper [5]. The isolation of volatile compounds is quite complex because honey contains a number of components with various chemical structures, polarity, and concentrations in a complex sugar matrix [6,7]. Many techniques have been applied for the isolation of volatile compounds from honey. Hydro-distillation (HD) [8,9]; microsimultaneous steam distillation–solvent extraction (MSDE) [9]; liquid-liquid extraction (LLE) [10,11]; dehydration homogeneous liquid-liquid extraction (DLLME) [11]; solid-phase extraction (SPE) [10,12]; dynamic headspace extraction (DHS) [13,14,15]; solid-phase microextraction (SPME) [16,17]; and ultrasound-assisted extraction (USE) [11,18] are mentioned in the literature. Among the above techniques, SPME is a well-accepted methodology because it’s simpler with no pre-treatment of samples and it is free of organic solvents. Different SPME fiber coatings are available, and the extraction is usually followed by gas chromatography-mass spectrometry (GC-MS), allowing the qualitative and quantitative determination of honey volatile fractions [19,20].

It is well known that several factors including temperature, equilibration time, extraction time, sample volume, water-honey ratio, and sodium chloride addition, affect volatiles’ isolation. However, optimum magnetic stirring velocities have not been studied yet. In the last decade, only a few studies [14,21,22,23,24] focused on optimizing the conditions for isolation of honey volatiles. In most studies, the procedure is followed by a one-factor-at-a-time technique or grouped factors to reduce the experimental design runs. To overcome this problem, the optimization can be carried out by using multivariate statistic techniques [25]. Symmetrical experimental designs (Box–Behnken, Doehlert designs, three-level factorial, and central composite) could be used to generate optimization models.

In this context, the aim of the present work was (a) to develop an optimized methodology of the SPME technique for the isolation of dominant volatile compounds of thyme honey using response surface methodology (RSM) (b) to evaluate the robustness of the model using response data validation from eighty unifloral Greek thyme honeys and (c) to optimize the magnetic stirrer velocity.

## 2. Results and Discussion

### 2.1. Physicochemical and Melissopalynological Analysis

The results of physicochemical and melissopalynological analysis agree with the botanical origin of the honey samples, as has been stated by the producers. Table 1 summarizes the results of physicochemical and melissopalynological analyses.

### 2.2. Isolation of Volatile Compounds

Table 2 presents the volatile compounds of thyme honey based on design layout. The identified compounds were 31 including esters, aldehydes, alcohols, ketones, hydrocarbons, nitriles, terpenes, and others.

**Figure 1:**
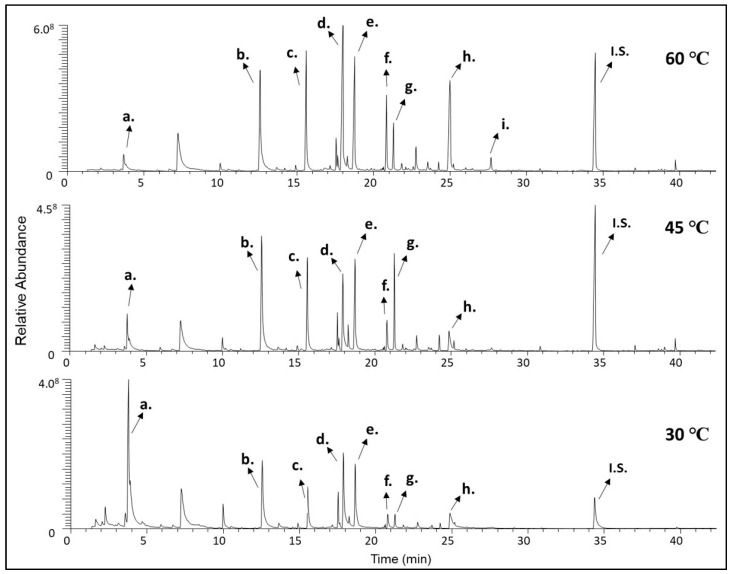
Characteristic chromatograms of the same sample from three representative different temperatures (30, 45, and 60 °C). (a) 3-methylbutanenitrile, (b) benzaldehyde, (c) 2-phenylacetaldehyde, (d) 2-phenylethan-1-ol, (e) 2-phenylacetonitrile, (f) 1-phenylbutane-2,3-dione, (g) methyl nonanoate, (h) 3-hydroxy-4-phenyl-2-butanone, (i) (*Z*)-3-hydroxy-4-phenylbut-3-en-2-one, (I.S.) Internal Standard.

Overall, the main compounds were 3-methylbutanenitrile, benzaldehyde, 2-phenylacetaldehyde, 2-phenylethan-1-ol, 2-phenylacetonitrile, 1-phenylbutane-2,3-dione, methyl nonanoate, 3-hydroxy-4-phenyl-2-butanone, and (*Z*)-3-hydroxy-4-phenylbut-3-en-2-one. Characteristic chromatograms from three different temperatures (30, 45, and 60 °C) are presented in Figure 1.

Summarizing the recent literature, volatile compounds of honey are strongly related to its botanical origin and the geographical area of collection. For this reason, a major criterion before optimization was to ensure the samples are monofloral. Our results have shown several similarities, comparing with other studies. Alissandrakis et al., [16] reported 1-phenylbutane-2,3-dione, 3-hydroxy-1-phenylbutan-2-one, 3-hydroxy-4-phenylbutan-2-one, 2-phenylacetonitrile, and 5-isopropyl-2-methylphenol in 28 monofloral honey samples from Greece. In a later study, Karabagias et al., [27] reported 2-phenylacetaldehyde, ethyl nonanoate, benzaldehyde, and 2-phenylethan-1-ol as dominant volatile compounds in 42 monofloral thyme honey samples from Greece. Four years later Karabagias et al., [28] reported 2-phenylacetaldehyde, 2-phenylacetonitrile, benzaldehyde, and 2-furancarboxaldehyde as characteristic volatile compounds in 31 thyme honey samples.

Honey volatile compounds originate in the floral source, while also precursors are converted during honey maturation [29]. In addition, honey composition can be affected by storage conditions [13,30], postharvest processing, and beekeepers’ manipulations [5]. Therefore, a careful selection of volatile compounds should be made when these are used in chemometric models. Considering the above, the dominant volatile compounds of monofloral Greek thyme honey were selected, excluding postharvest generated compounds such as furans. Each volatile response was used for the construction of the optimization model based on RSM.

### 2.3. Evaluation of Factors

Due to variation of factors, these were coded as temperature (°C) (A); equilibration time (min) (B); extraction time (min) (C); magnetic stirrer speed (rpm) (D); sample volume (mL) (E); water: honey (*v*/*w*) (F).

**Figure 2:**
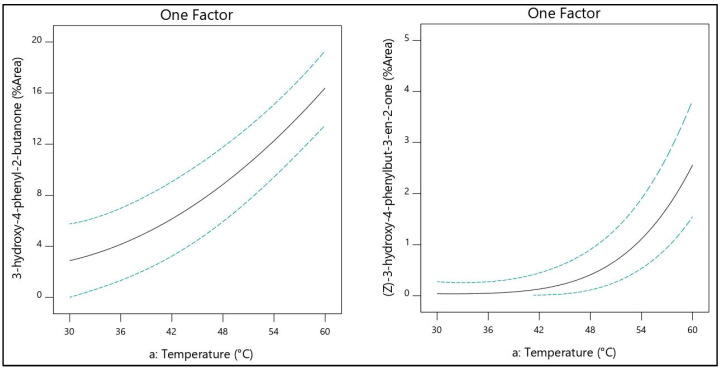
Temperature effect in the isolation of 3-hydroxy-4-phenyl-2-butanone, and (*Z*)-3-hydroxy-4-phenylbut-3-en-2-one.

The temperature showed a significant effect on volatile recovery. Setting 60 °C as an upper limit diminished the probability of losing compounds due to thermal desorption from fiber and avoiding at the same time the formication of by-products. As it is seen in Table 2 the dominant volatile compounds of thyme honey present high boiling points in most as a measure of their volatility. Characteristically, the isolation of 3-hydroxy-4-phenyl-2-butanone, and (*Z*)-3-hydroxy-4-phenylbut-3-en-2-one increased exponentially with temperature as shown in Figure 2.

Equilibration time does not influence significantly the volatile isolation. However, this factor ensures the repeatability of volatiles qualitative and quantitative determination [21] so it should be taken into account. The above remark agrees with the results of this study based on the determination of the coefficient (*p* < 0.05).

The extraction time was a significant parameter with a positive influence on most of the volatile compounds. The obtained results indicate that extraction time under 30 min didn’t achieve a state of equilibrium while over 30 min saturation of the fiber occurred leading to reduced adsorption of volatiles. Also, at 60 min extraction time lower isolation efficiency of compounds with high volatility was observed. As previously remarked by Plutowska et al., [21], compounds with short equilibration time can be displaced gradually from the fiber and counterbalanced by compounds with lower volatility. Therefore, depending on the nature of the honey and the purpose of the study the extraction time can be varied.

The magnetic stirrer speed is one factor that has not been evaluated so far. Stirring accelerates mass transfer between phases and the establishing phase equilibrium allowing better isolation of compounds with lower volatility. Predictive models were created to evaluate the magnetic stirrer speed and the correlation with other factors. The obtained results indicate that the investigated range of magnetic stirrer speed, for dominant volatile compounds, presented a state of relative impact by comparing the factor coefficients. So, it was seen that benzaldehyde presented positive effect by D, BD, CD, DF, D^2^, 2-phenylacetaldehyde by BD, DF, undecane by AD, BD, CD, DF, nonanal by D, AD, 2-phenylethan-1-ol by BD, 2-phenylacetonitrile by BD, D^2^, 1-phenylbutane-2,3-dione by D, AD, BD, D^2^, methyl nonanoate by D, AD, CD, DE, 3-hydroxy-4-phenyl-2-butanone by DE, D^2^, and (*Z*)-3-hydroxy-4-phenylbut-3-en-2-one by D^2^. However, these terms are predictive and they cannot be used for modeling future responses but can be used to re-create the results of this experiment. In each case, the speed of the magnetic stirrer should be taken into account.

In some cases, while increasing the sample volume, in a stable vial, the SPME fiber adsorbed more volatile compounds and then remaining relatively constant [31]. Equilibrium is attained more rapidly in the headspace of the vial and volatiles can diffuse more quickly and efficiently to the coating on the fiber [32]. However, in this study, increasing the sample volume did not confirmed the above case. This factor didn’t have a significant impact on the isolation of volatiles except for undecane and methyl nonanoate (*p* < 0.05). Another study also reported that the efficiency of isolation is the same for all volumes examined [21].

**Figure 3:**
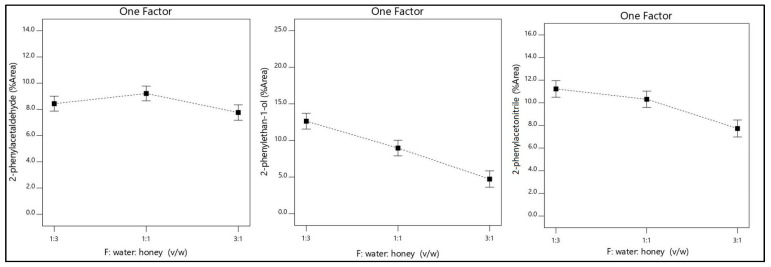
The ratio of water: honey (*v*/*w*) effect in the isolation of 2-phenylacetaldehyde, 2-phenylethan-1-ol, and 2-phenylacetonitrile.

**Figure 4:**
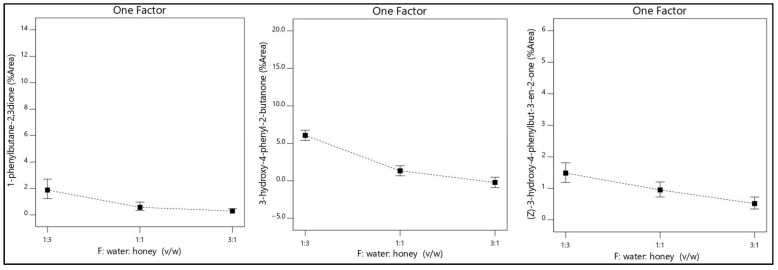
The ratio of water: honey (*v*/*w*) effect in the isolation of 1-phenylbutane-2,3-dione, 3-hydroxy-4-phenyl-2-butanone, and (*Z*)-3-hydroxy-4-phenylbut-3-en-2-one.

The ratio of water: honey (*v*/*w*) was considered as important as the temperature for all responses. Dilution of the honey sample with water in specific proportions in contrast with undiluted honey increased the isolation of the major volatile compounds. Also, it has been reported high difficulty to acquire satisfactory repeatability of isolation using undiluted honey [21]. On the other hand, the addition of a large amount of water reduces isolation efficacy as shown in Figure 3 and Figure 4.

### 2.4. Evaluation and Optimization of Dominant Volatile Compounds

A total of 10 responses (volatile compounds) were used as data for optimization (Table 3).

It was confirmed each response follows the normal distribution, and diagnostic tests of Box-Cox were included in Figures S1–S10. Also, data were evaluated via the determination of coefficient (R^2^). The ANOVA, in this case, confirms the adequacy of the model (*p*-value is less than 0.05) and indicates model terms are significant. Table 4 present a summary coefficient for each response subjected to the model.

The volatility of some responses also depends on combinations of the independent factors. Benzaldehyde contingent on AC, EF, A^2^, 2-phenylacetaldehyde on AC, AF, A^2^, undecane on AD, AE, AF, A^2^, 2-phenylacetonitrile on AB, A^2^, methyl nonanoate on AB, CF, and 3-hydroxy-4-phenyl-2-butanone on AF, A^2^. Nonanal, 2-phenylethan-1-ol, 1-phenylbutane-2,3-dione, and (*Z*)-3-hydroxy-4-phenylbut-3-en-2-one depend on individual factors.

Before developing an optimization model that is based on the combination of volatile compounds, it was deemed useful to estimate the optimum conditions for each volatile. All results were evaluated by desirability indices. Desirabilities range from zero to one for any given volatile compound. Zero value indicates that volatile compounds fall outside desirable limits and one value represents the ideal case. Optimum conditions, desirabilities, and predicted mean for each response are presented in Table 5.

**Figure 5:**
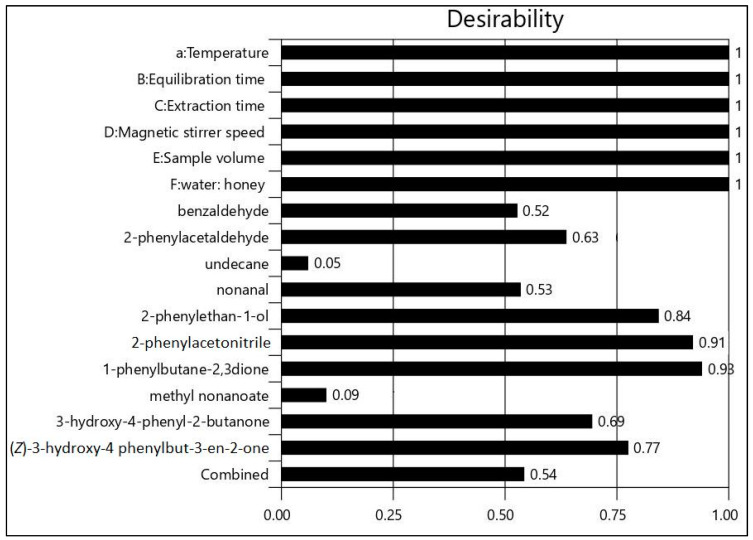
Desirabilities of overall volatile compound responses based on optimum isolation conditions.

The best conditions proposed for the overall response of volatile components was A: 60 °C, B: 15 min, C: 30 min, D: 700 rpm, E: 6 mL, F: 1:3 (*v*/*w*) with total desirability over 0.50 (Figure 5). Predicted mean was calculated for benzaldehyde (11.3%), 2-phenylacetaldehyde (8.9%), undecane (1.5%), nonanal (1.4%), 2-phenylethan-1-ol (14.1%), 2-phenylacetonitrile (10.8%), 1-phenylbutane-2,3-dione (6.0%), methyl nonanoate (2.0%), 3-hydroxy-4-phenyl-2-butanone (12.0%), (*Z*)-3-hydroxy-4-phenylbut-3-en-2-one (1.0%).

A test set of 80 monofloral thyme samples was submitted for validation of the optimization model and their quantitative analysis are presented in Table S1. These samples were analyzed under optimum conditions of SPME using GC-MS. Dominant volatile compounds were isolated in all validation samples. Confirmation data mean calculated for benzaldehyde (7.1%), 2-phenylacetaldehyde (20.9%), undecane (1.0%), nonanal (3.8%), 2-phenylethan-1-ol (4.9%), 2-phenylacetonitrile (5.8%), 1-phenylbutane-2,3-dione (8.2%), methyl nonanoate (2.3%), 3-hydroxy-4-phenyl-2-butanone (9.6%), (*Z*)-3-hydroxy-4-phenylbut-3-en-2-one (3.2%). Most results, based on the developed RSM models, confirm that they operate at the 95% prediction interval. However, due to the nature of honey and its variability as food, we should be wary and more tolerant of the results of the prediction interval. Nevertheless, the total results are satisfactory and the above RSM model could provide suitable conditions for the isolation of dominant volatile compounds for Greek monofloral thyme honey.

## 3. Materials and Methods

### 3.1. Honey Samples

A total of 81 monofloral thyme honey samples were obtained during the 2019–2020 harvest years. The botanical origin was first assessed by the beekeepers and confirmed after physiochemical [33,34] and melissopalynological [35] analysis as previously described [36]. Honey samples were stored in hermetically closed glass bottles and kept in the dark at 4 °C until further analysis.

### 3.2. Experimental Design

The RSM methodology was developed by Box and Wilson [37] with notable applications in the design, development, and formulation of new experimental models, or improvement of existing experimental data. RSM consists of a collection of mathematical and statistical techniques that is useful for the approximation and optimization of experimental data sets, and its use has been widely adopted in chemometrics too [38,39]. Practically, through a polynomial equation, an attempt is established to maximize the responses under the influence of many independent factors. Towards this objective, to optimize the isolation of volatile compounds by SPME, a central composite design (CCD) was used in which five numeric factors and one categorical independent factor were analyzed: temperature (°C) (A); equilibration time (min) (B); extraction time (min) (C); magnetic stirrer speed (rpm) (D); sample volume (mL) (E); water: honey (*v*/*w*) (F). A quadratic design model was performed under five groups and 38 runs, as they were calculated from the experimental design. A randomly selected thyme honey sample was used for response prediction and 80 samples were used as confirmation response data for the robustness of the model. More detailed information about independent experimental factors and design layout runs are shown in Table 6 and Table S2 respectively.

The evaluation of the model’s fitness was confirmed using the *p*-values through an analysis of variance (ANOVA) and the determination coefficient (R^2^). Volatile compounds (dependent variables) also called and as responses were validated in terms of the evaluation of Box-Cox test, correlations, and normality of residuals. Then, for each response optimization criteria or constraints were set including factors and propagation of error. The goal was to construct maximize desirability indices and confirm all possible solutions by numerical and graphical plots. Finally, the model was confirmed with volatile compounds (response data) from SPME analysis of eighty thyme honey samples according to the optimum solution.

The desirability and response surface were performed with statistical program Design-Expert 11.0.5.0 (Stat-Ease, Inc., Minneapolis, MN, USA).

### 3.3. Isolation and Analysis of Volatile Compounds

All optimization experiments were performed using a manual holder with triple-phase divinylbenzene/carboxen/polydimethylsiloxane (DVB/CAR/PDMS) fiber 50/30 μm (Supelco, Bellefonte, PA, USA) with a length of 1 cm. Before isolation, all fibers were conditioned at 270 °C. Based on each experimental design layout run (Table S2), thyme honey was diluted with water and a predetermined volume ratio of water: honey (*v*/*w*) was adopted in 15 mL screw top (22.7 × 86 mm), vials with PTFE/silicone septa. A portion of 20 μL (300 μg mL^−1^ in methanol) of benzophenone (Alfa Aesar, Kandel, Germany) was used as an internal standard.

The analysis of volatile compounds was performed using a Trace Ultra gas chromatograph (GC) (Thermo Scientific Inc., Waltham, MA, USA), coupled to a mass spectrometer (MS) (DSQII, Thermo Scientific Inc., Waltham, MA, USA) as described previously [17]. More detailed, the column used was a Restek Rtx-5MS (30 m × 0.25 mm i.d., 0.25 µm film thickness) and the carrier gas was helium at a 1 mL min^−1^ rate. The desorption conditions were as follows: GC inlet temperature 260 °C in the splitless mode for 3 min, with a 0.8 mm injector liner (SGE International Pty Ltd., Ringwood, Australia). Oven temperature was adapted to 40 °C for 6 min, then increased at 120 °C at a rate of 5 °C min^−1^, followed by an increment of 3 °C min^−1^ up to 160 °C and up to 250 °C with a step of 15 °C min^−1^. Finally, the temperature of 250 °C was kept constant for 1 min. The transfer line and injector temperatures were maintained at 290 and 220 °C, respectively. Electron impact was 70 eV, and mass spectra were recorded at the 35–650 mass range. Before each analysis, a blank sample were performed (Figure S11). The peak identification was carried out with the Wiley 275 mass spectra library, its masses spectral data, and the arithmetic index provided by Adams [40]. Retention Index (RI) values of volatile compounds were calculated using n-alkane (C8–C20) standards (Supelco, Bellefonte, PA, USA). The isolated compounds were semi-quantified against the internal standard (benzophenone) and expressed as mg/kg of honey.

## 4. Conclusions

According to the experimental design, we concluded that the proposed chemometric methodology is well-suited for the optimization of the isolation of volatile compounds from monofloral thyme honey. SPME-GC-MS in combination with RSM led to precognition of the optimum conditions (A: 60 °C, B: 15 min, C: 30 min, D: 700 rpm, E: 6 mL, F: 1:3 (*v*/*w*)), desirabilities, and predicted mean for each volatile response. The temperature, extraction time and ratio of water: honey (*v*/*w*) were the most significant factors with a positive impact on the recovery for most of the volatile compounds. Equilibration time ensured the qualitative and quantitative repeatability, while sample volume was related to the isolation of minor compounds. Although the effect of magnetic stirrer velocity has not been evaluated as a factor affecting extraction efficacy, it was found to be quite important under predictive models, especially when combined with other factors. In some cases, magnetic stirrer speed can act synergistically in the optimization of responses. As it emerged by evaluation of factors, the optimum conditions of some of them depend on the nature of the honey. In addition, a test set of 80 monofloral thyme honey validated these results and reinforced the proposed optimization model. It is useful to remark that this methodology can be applied to highlight the dominant volatiles of thyme honey produced from Greek *Thymbra capitata* L., thereby making known their extraordinary aroma profile. As arisen from the 80 validation test samples, the proposed optimization methodology increased the sensitivity of isolated volatile compounds in combination with the rapidity of the method. This study could be a good basis for the development of novel RSM for other monofloral honey samples that contain volatile compounds that belong to different categories of chemical compounds with different properties and volatility, and probably different optimum conditions.

## Figures and Tables

**Table 1:** Physicochemical and melissopalynological properties of samples used.

Aggregate Functions	Fructose + Glucose ^a^ (%*w*/*w*)	Sucrose ^b^ (%*w*/*w*)	Moisture ^c^ (%*w*/*w*)	Electrical Conductivity ^d^ (μS cm^−1^)	Diastase Activity ^e^ (Schade)	HMF ^f^ (mg kg^−1^)	*Thymbra capitata* L. Pollen ^g^ (%)
Min	60.1	0.0	13.8	251	11.1	1.0	18.0
Max	86.4	2.1	17.9	600	51.1	14.7	77.9
Average	68.1	0.3	15.8	457	27.3	5.2	33.1

According to the Greek legislation [26]: ^a^ Sum of fructose and glucose not less than 60 (%*w*/*w*); ^b^ Sucrose content not more than 5 (%*w*/*w*); ^c^ Moisture content not more than 20 (%*w*/*w*); ^d^ Electrical conductivity not more than 600 (μS cm^−1^); ^e^ Diastase activity not less than 8 Schade; ^f^ HMF not more than 40 mg kg^−1^; ^g^ *Thymbra capitata* L. pollen not less than 18% and absence of a species with not more than 45%.

**Table 2:** Volatile compounds isolated from the headspace of thyme honey.

No.	Volatile Compounds	RT ^a^	RI ^b^	Boiling Point (°C)	Min (mg kg^−1^)	Max (mg kg^−1^)	Average (mg kg^−1^)
	**Esters**						
1	methyl octanoate	18.3	1123	190.6	0.00	2.72	0.46
2	methyl nonanoate	21.3	1222	213.5	0.09	11.63	1.41
3	methyl decanoate	24.3	1321	236.4	0.00	1.17	0.12
4	methyl hexadecanoate	39.7	1929	373.6	0.00	0.46	0.07
	**Aldehydes**						
5	furan-2-carbaldehyde	9.8	822	139.7	0.20	4.54	1.90
6	benzaldehyde	12.6	957	162.0	0.52	10.40	3.64
7	2-phenylacetaldehyde	15.6	1041	184.8	0.39	3.56	1.78
8	nonanal	17.7	1104	181.0	0.00	1.45	0.31
9	decanal	20.8	1205	203.9	0.00	1.41	0.21
10	4-isopropylbenzaldehyde	21.8	1240	235.1	0.00	0.20	0.05
	**Alcohols**						
11	2-phenylethan-1-ol	17.9	1111	228.4	0.00	5.28	1.94
12	5-isopropyl-2-methylphenol (carvacrol)	23.5	1299	267.1	0.00	0.26	0.06
	**Ketones**						
13	1-phenylbutane-2,3-dione	20.9	1210	289.7	0.00	1.37	0.48
14	2-isopropyl-5-methylcyclohexa-2,5-diene-1,4-dione (thymoquinone)	22.0	1247	323.0	0.00	0.59	0.08
15	3-hydroxy-4-phenyl-2-butanone	24.9	1343	327.5	0.00	3.87	0.93
16	(*Z*)-3-hydroxy-4-phenylbut-3-en-2-one	27.7	1427	332.0	0.00	0.37	0.09
	**Hydrocarbons**						
17	butane	1.6	<800	18.0	0.00	20.58	1.03
18	heptane	3.4	<800	86.6	0.00	10.32	0.58
19	octane	5.9	800	109.5	0.00	5.83	0.45
20	nonane	10.3	896	132.4	0.00	1.68	0.20
21	undecane	17.6	1100	178.1	0.04	34.49	2.33
	**Nitriles**						
22	isobutyronitrile	2.2	<800	119.6	0.00	11.66	0.92
23	2-methylbutanenitrile	3.5	<800	142.5	0.00	5.01	0.47
24	3-methylbutanenitrile	3.7	<800	142.5	0.05	59.16	7.01
25	2-phenylacetonitrile	18.8	1136	215.4	0.25	5.35	1.93
	**Terpenoids**						
26	methylbenzene	4.7	<800	113.3	0.00	3.38	0.34
27	1-isopropyl-4-methylbenzene (p-cymene)	14.9	1022	186.5	0.00	2.62	0.39
28	1-methyl-4-(prop-1-en-2-yl)benzene	17.2	1088	183.5	0.00	1.14	0.17
	**Others**						
29	2,5-diethyltetrahydrofuran	10.0	890	147.1	0.00	7.22	1.35
30	methyl 2-oxo-2-phenylacetate	22.7	1271	271.1	0.00	0.54	0.18
31	1,1,5-trimethyl-1,2-dihydronaphthalene	25.2	1351	270.9	0.00	1.39	0.37

^a^ RT: Retention time (min); ^b^ RI: Experimental retention index.

**Table 3:** Responses (volatile compounds) used for the optimization model.

Response	Volatile Compound	Min (%Area)	Max (%Area)	Mean (%Area)	Std. Dev.
R1	benzaldehyde	4.60	17.32	11.55	1.57
R2	2-phenylacetaldehyde	0.82	13.48	6.99	1.03
R3	undecane	0.62	16.17	3.86	0.85
R4	nonanal	0.00	2.62	0.99	0.38
R5	2-phenylethan-1-ol	0.00	16.72	8.12	1.94
R6	2-phenylacetonitrile	1.40	11.59	6.97	1.31
R7	1-phenylbutane-2,3-dione	0.00	6.49	1.22	0.52
R8	methyl nonanoate	0.53	15.23	4.32	2.27
R9	3-hydroxy-4-phenyl-2-butanone	0.00	17.26	4.58	1.22
R10	(*Z*)-3-hydroxy-4-phenylbut-3-en-2-one	0.00	1.64	0.45	0.11

**Table 4:** Coefficients and R^2^ of each response subjected to the model.

Response	Volatile Compound	A	B	C	D	E	F	R^2^
R1	benzaldehyde	0.011 ^a^	0.198	0.101	0.259	0.439	0.007	0.965
R2	2-phenylacetaldehyde	0.000	0.265	0.657	0.898	0.104	0.059	0.988
R3	undecane	0.000	0.319	0.001	0.010	0.013	0.001	0.991
R4	nonanal	0.090	0.951	0.843	0.004	0.168	0.007	0.958
R5	2-phenylethan-1-ol	0.000	0.545	0.013	0.170	0.324	0.001	0.980
R6	2-phenylacetonitrile	0.002	0.587	0.018	0.724	0.161	0.005	0.965
R7	1-phenylbutane-2,3-dione	0.001	0.943	0.078	0.916	0.413	0.012	0.962
R8	methyl nonanoate	0.179	0.697	0.364	0.246	0.026	0.031	0.932
R9	3-hydroxy-4-phenyl-2-butanone	0.000	0.656	0.001	0.175	0.542	0.000	0.991
R10	(*Z*)-3-hydroxy-4-phenylbut-3-en-2-one	0.014	0.092	0.011	0.680	0.357	0.026	0.994

^a^ (*p*-value < 0.05).

**Table 5:** Optimum conditions, desirabilities, and predicted mean for each response.

Response	Volatile Compound	A	B	C	D	E	F	Desirability	Predicted Mean (%Area)
R1	benzaldehyde	60	5	15	700	6	1:1	0.810	14.9 ± 1.6
R2	2-phenylacetaldehyde	60	15	15	400	6	1:1	0.975	13.2 ± 1.0
R3	undecane	30	15	15	400	2	3:1	0.940	15.2 ± 0.9
R4	nonanal	45	15	30	700	4	3:1	0.933	2.4 ± 0.4
R5	2-phenylethan-1-ol	60	15	15	400	6	1:3	0.923	15.4 ± 1.9
R6	2-phenylacetonitrile	60	15	30	400	6	1:3	0.957	11.1 ± 1.3
R7	1-phenylbutane-2.3dione	60	30	30	700	6	1:3	0.966	6.3 ± 0.6
R8	methyl nonanoate	60	30	30	400	2	3:1	0.814	12.5 ± 2.3
R9	3-hydroxy-4-phenyl-2-butanone	60	30	30	700	4	1:3	0.841	14.5 ± 1.2
R10	(*Z*)-3-hydroxy-4-phenylbut-3-en-2-one	60	5	30	700	6	1:3	0.947	1.5 ± 0.3

**Table 6:** Independent experimental factors.

Factor	Name	Units	Minimum	Maximum	Coded Low	Coded High	Mean	Std. Dev.
A	Temperature	°C	30.0	60.0	−1 ↔ 30.0	+1 ↔ 60.0	45.0	13.5
B	Equilibration time	min	5.0	30.0	−1 ↔ 5.0	+1 ↔ 30.0	17.7	11.6
C	Extraction time	min	15.0	60.0	−1 ↔ 15.0	+1 ↔ 60.0	36.7	20.8
D	Magnetic stirrer speed	rpm	100.0	700.0	−1 ↔ 100.0	+1 ↔ 700.0	407.8	283.2
E	Sample volume	mL	2.0	6.0	−1 ↔ 2.0	+1 ↔ 6.0	4.1	1.8
F	Water:honey ratio	*v*/*w*	1:3	3:1				

## Data Availability

Not applicable.

## Supplementary Materials

The following are available online. Figure S1: Box-cox plot of benzaldehyde. Figure S2: Box-cox plot of 2-phenylacetaldehyde. Figure S3: Box-cox plot of undecane. Figure S4: Box-cox plot of nonanal. Figure S5: Box-cox plot of 2-phenylethan-1-ol. Figure S6: Box-cox plot of 2-phenylacetonitrile. Figure S7: Box-cox plot of 1-phenylbutane-2.3dione. Figure S8: Box-cox plot of methyl nonanoate. Figure S9: Box-cox plot of 3-hydroxy-4-phenyl-2-butanone. Figure S10: Box-cox plot of (*Z*)-3-hydroxy-4-phenylbut-3-en-2-one. Figure S11. A characteristic chromatograph from blank sample. Table S1: Volatile compounds isolated from the headspace of thyme honey samples used for validation test. Table S2: Design layout runs.
