# Supplementary material for: Response Surface Methodology to Optimize the Isolation of Dominant Volatile Compounds from Monofloral Greek Thyme Honey Using SPME-GC-MS
Source: Molecules. 2021 Jun 12;26(12):3612. doi: 10.3390/molecules26123612 (PMC8231491; doi:10.3390/molecules26123612)
Supplement: Supplementary file 1 [file molecules-26-03612-s001.zip › molecules-1250670-supplementary.pdf]

Supplementary Materials

Response Surface Methodology to Optimize the Isolation of Dominant Volatile Compounds from Monofloral Greek Thyme Honey using SPME-GC-MS

Marinos Xagoraris [1], Alexandra Skouria [1], Panagiota-Kyriaki Revelou [1], Eleftherios Alissandrakis [2], Petros A. Tarantilis [1] and Christos S. Pappas [1,*]

[1] Laboratory of Chemistry, Department of Food Science and Human Nutrition, Agricultural University of Athens, 75 Iera Odos, 11855 Athens, Greece; mxagor@aua.gr (M.X); stud15080@aua.gr (A.S.); p.revelou@aua.gr (P.-K.R.); ptara@aua.gr (P.A.T.)

[2] Laboratory of Quality and Safety of Agricultural Products, Landscape and Environment, Department of Agriculture, Hellenic Mediterranean University, Stavromenos, Heraklion, PC 71410 Crete, Greece; ealiss@hmu.gr (E.A.)

* Correspondence: chrispap@aua.gr; Tel. +30-2105294262.

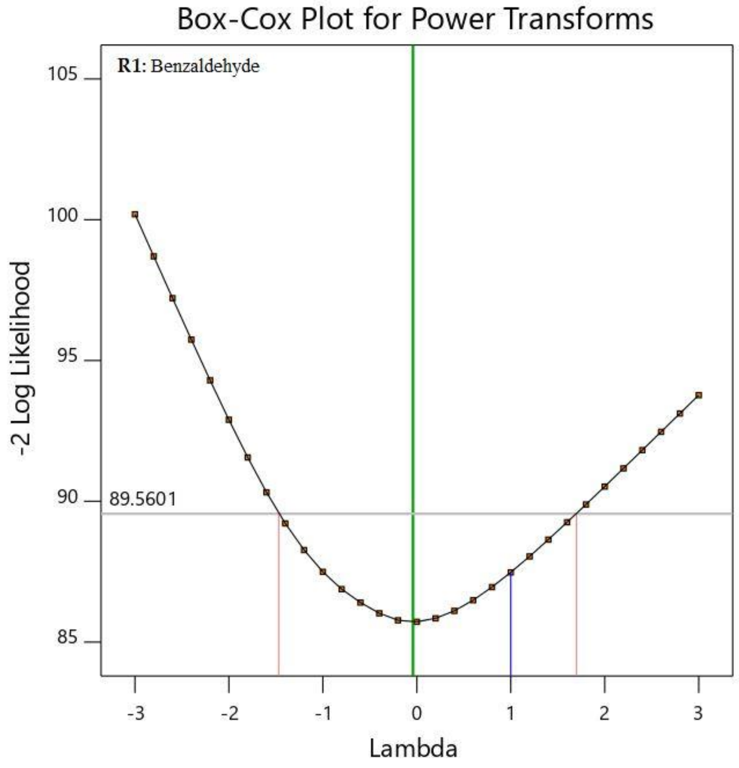

Figure S1. Box-cox plot of benzaldehyde.

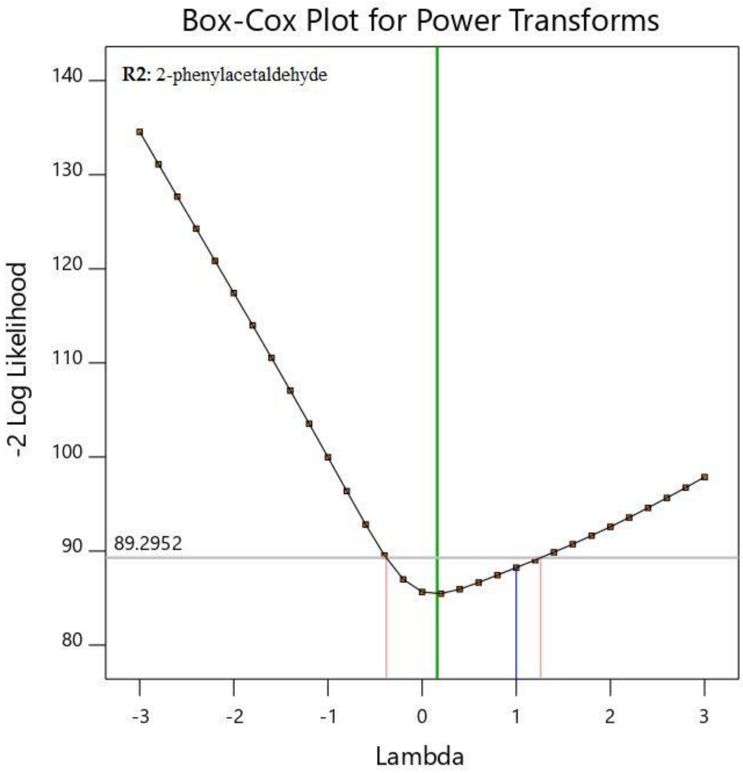

Figure S2. Box-cox plot of 2-phenylacetaldehyde.

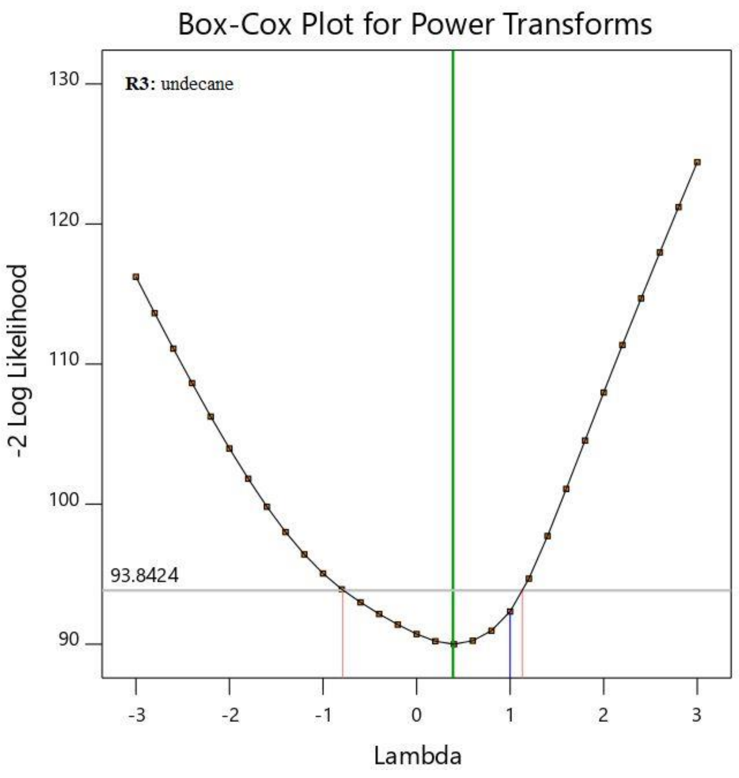

Figure S3. Box-cox plot of undecane.

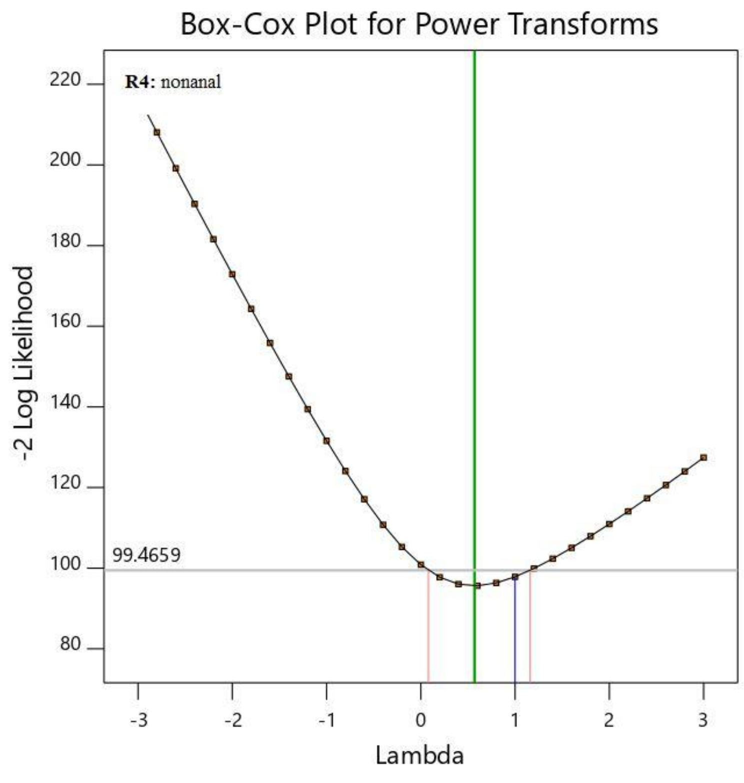

Figure S4. Box-cox plot of nonanal.

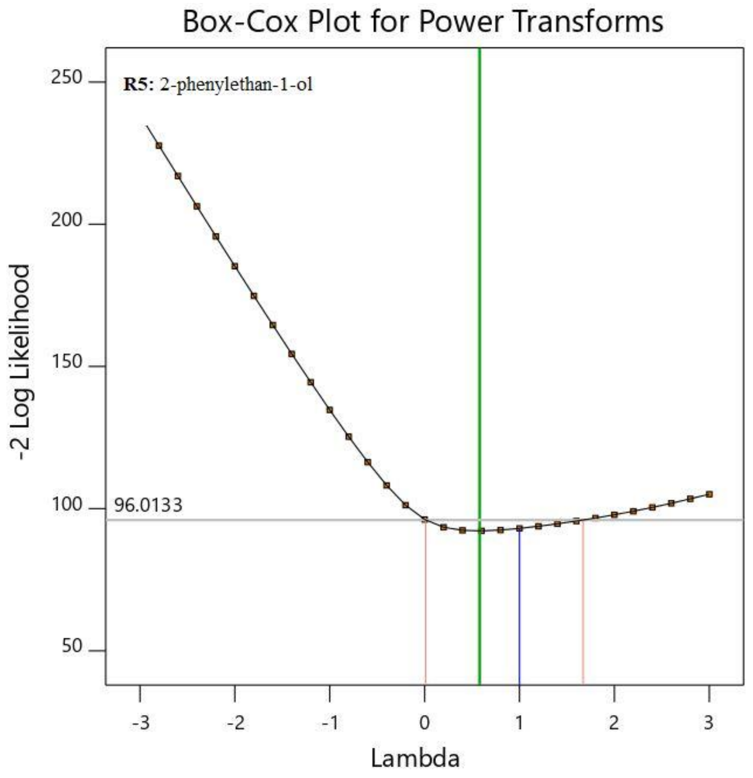

Figure S5. Box-cox plot of 2-phenylethan-1-ol.

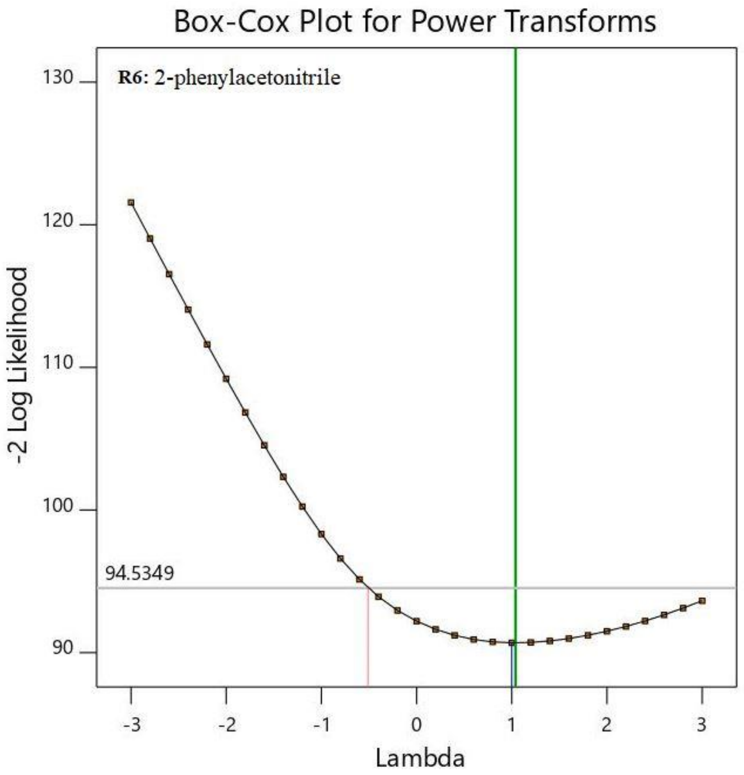

Figure S6. Box-cox plot of 2-phenylacetonitrile.

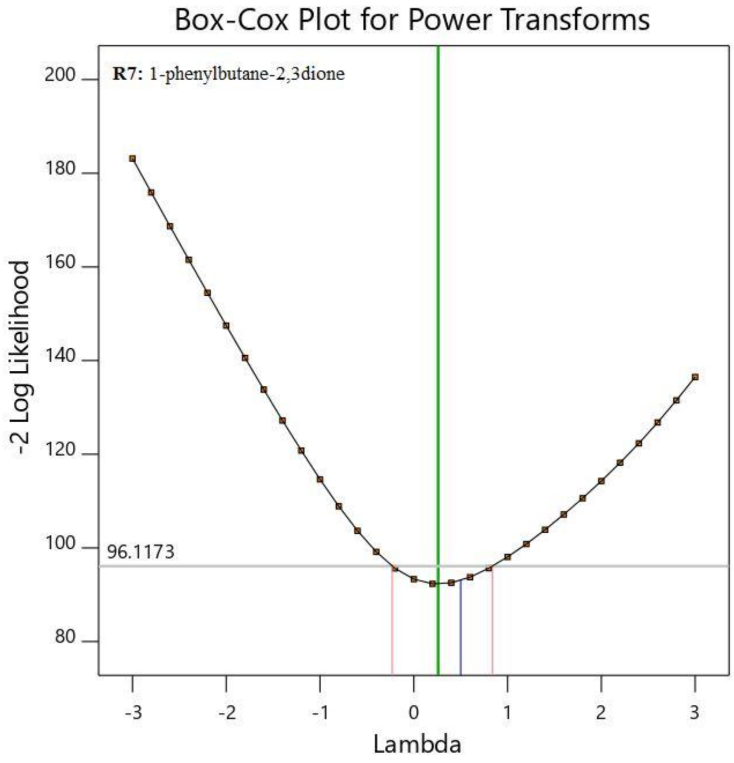

Figure S7. Box-cox plot of 1-phenylbutane-2.3dione.

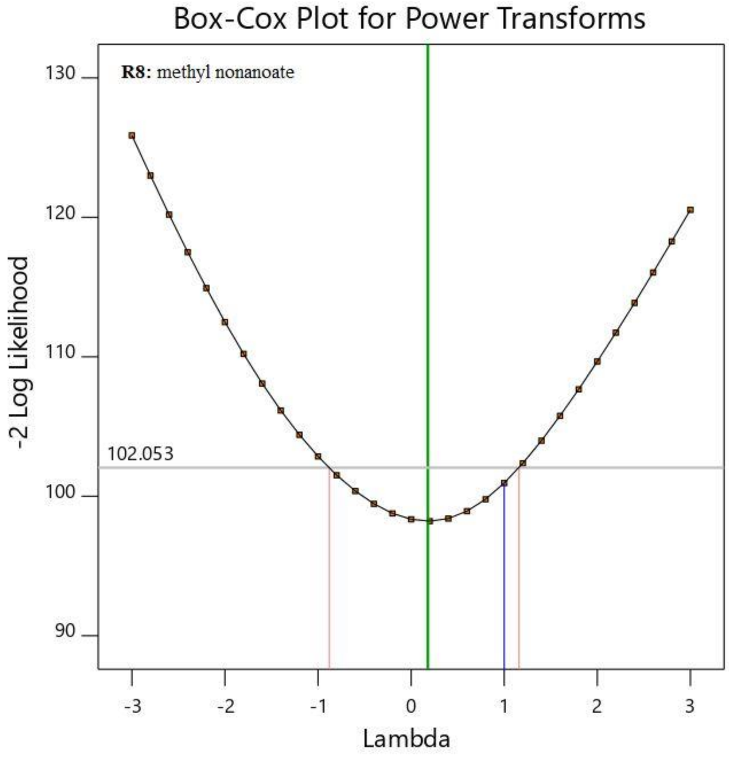

Figure S8. Box-cox plot of methyl nonanoate.

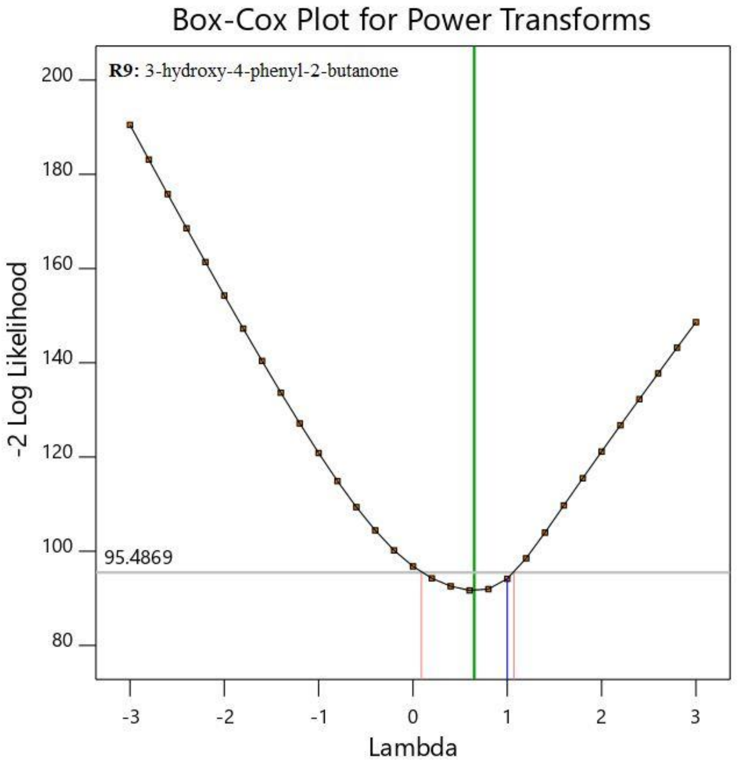

Figure S9. Box-cox plot of 3-hydroxy-4-phenyl-2-butanone.

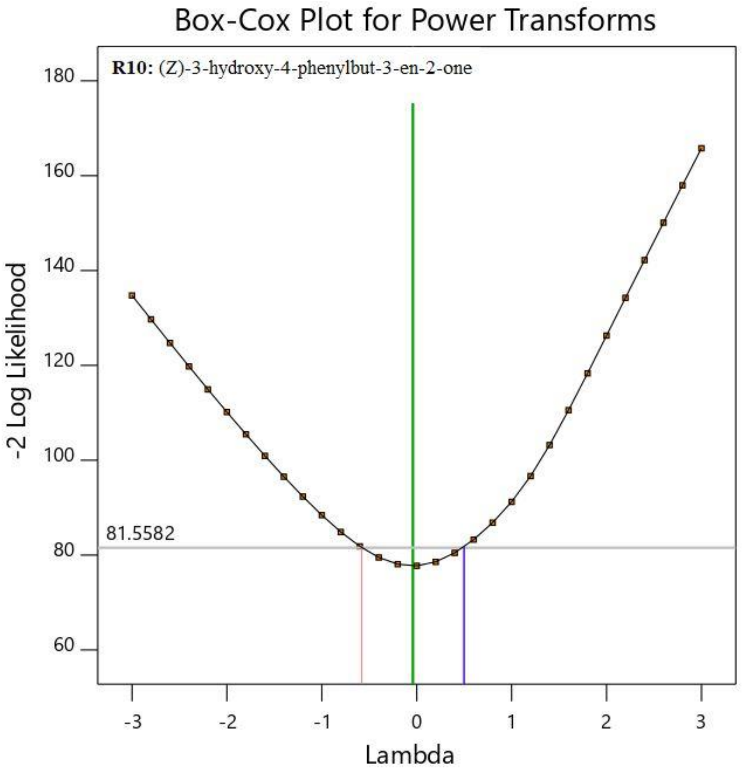

Figure S10. Box-cox plot of (*Z*)-3-hydroxy-4-phenylbut-3-en-2-one.

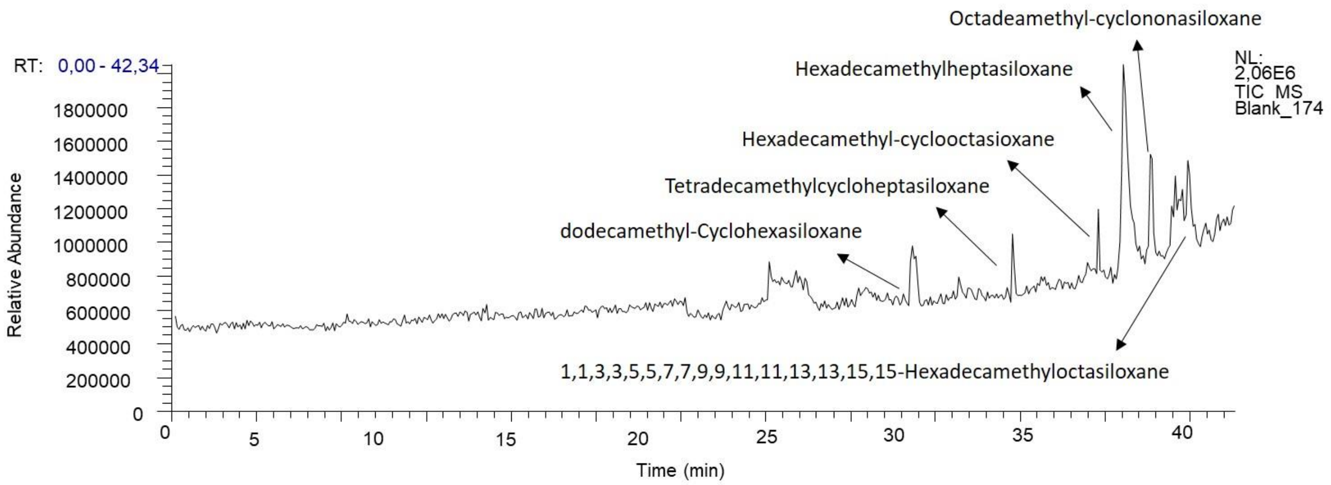

Figure S11. A characteristic chromatograph from blank sample.

Table S1. Volatile compounds isolated from the headspace of thyme honey samples used for validation test.

No.	Volatile compounds	RT[a]	RI[b]	Boiling point (°C)	Min (mg kg^{-1})	Max (mg kg^{-1})	Average (mg kg^{-1})
			**Esters**				
1	methyl octanoate	18.3	1123	190.6	0.00	0.28	0.07
2	methyl nonanoate	21.3	1222	213.5	0.00	0.62	0.21
3	methyl decanoate	24.3	1321	236.4	0.00	0.16	0.05
4	methyl hexadecanoate	39.7	1929	373.6	0.00	0.21	0.05
			**Aldehydes**				
5	furan-2-carbaldehyde	9.8	822	139.7	0.00	0.85	0.20
6	benzaldehyde	12.6	957	162.0	0.15	3.61	0.84
7	2-phenylacetaldehyde	15.6	1041	184.8	0.19	11.91	2.52
8	nonanal	17.7	1104	181.0	0.00	2.03	0.52
9	decanal	20.8	1205	203.9	0.00	0.87	0.11
10	4-isopropylbenzaldehyde	21.8	1240	235.1	0.00	0.27	0.04
			**Alcohols**				
11	2-phenylethan-1-ol	17.9	1111	228.4	0.00	2.03	0.52
12	5-isopropyl-2-methylphenol (carvacrol)	23.5	1299	267.1	0.00	0.30	0.05
			**Ketones**				
13	1-phenylbutane-2,3-dione	20.9	1210	289.7	0.00	6.10	1.38
14	2-isopropyl-5-methylcyclohexa-2,5-diene-1,4-dione (thymoquinone)	22.0	1247	323.0	0.00	0.16	0.01
15	3-hydroxy-4-phenyl-2-butanone	24.9	1343	327.5	0.00	3.49	1.14
16	(*Z*)-3-hydroxy-4-phenylbut-3-en-2-one	27.7	1427	332.0	0.00	2.23	0.47
			**Hydrocarbons**				
17	butane	1.6	<800	18.0	0.00	0.00	0.00
18	heptane	3.4	<800	86.6	0.00	0.00	0.00
19	octane	5.9	800	109.5	0.00	0.07	0.01
20	nonane	10.3	896	132.4	0.00	0.04	0.00
21	undecane	17.6	1100	178.1	0.00	0.32	0.10
			**Nitriles**				
22	isobutyronitrile	2.2	<800	119.6	0.00	0.08	0.00
23	2-methylbutanenitrile	3.5	<800	142.5	0.00	0.00	0.00
24	3-methylbutanenitrile	3.7	<800	142.5	0.03	1.28	0.07
25	2-phenylacetonitrile	18.8	1136	215.4	0.00	3.49	0.78
			**Terpenoids**				
26	methylbenzene	4.7	<800	113.3	0.00	0.00	0.00
27	1-isopropyl-4-methylbenzene (p-cymene)	14.9	1022	186.5	0.00	0.07	0.00
28	1-methyl-4-(prop-1-en-2-yl)benzene	17.2	1088	183.5	0.00	0.16	0.01
			**Others**				
29	2,5-diethyltetrahydrofuran	10.0	890	147.1	0.00	0.27	0.04
30	methyl 2-oxo-2-phenylacetate	22.7	1271	271.1	0.00	0.00	0.00
31	1,1,5-trimethyl-1,2-dihydronaphthalene	25.2	1351	270.9	0.00	0.62	0.04

Table S2. Design layout runs.

Run	A: Temperature	B: Equilibration time	C: Extraction time	D: Magnetic stirrer speed	E: Sample volume	F: Water: honey ratio
Units	°C	min	min	rpm	mL	v/w
1	30	30	30	400	6	1:3
2	30	5	15	700	4	1:3
3	30	5	60	400	2	1:1
4	30	30	60	100	2	1:3
5	30	5	15	100	6	1:1
6	30	30	60	700	6	1:1
7	30	15	15	100	4	3:1
8	30	15	60	700	6	1:3
9	30	30	60	100	6	3:1
10	30	5	30	700	6	3:1
11	30	30	15	700	6	3:1
12	30	30	30	100	4	1:1
13	30	30	60	700	2	3:1
14	30	15	15	700	2	1:1
15	30	5	15	100	2	1:3
16	60	5	60	100	2	3:1
17	60	30	60	100	6	1:1
18	60	5	15	100	6	3:1
19	60	30	30	700	4	3:1
20	60	15	30	400	2	1:3
21	60	30	15	100	2	1:1
22	60	5	60	700	6	1:1
23	60	5	30	100	2	1:1
24	60	30	15	700	6	1:1
25	60	30	60	700	6	1:3
26	60	30	60	700	2	1:1
27	60	30	15	100	4	1:3
28	60	5	15	700	6	1:3
29	60	5	60	400	6	3:1
30	60	5	15	700	2	3:1
31	45	5	60	100	6	1:3
32	45	30	15	100	6	1:3
33	45	15	60	100	2	1:1
34	45	5	60	700	2	1:3
35	45	30	30	100	2	3:1
36	45	30	15	700	2	1:3
37	45	5	15	400	4	1:1
38	45	15	60	700	6	3:1
